# Oxidative Stress: A Link between Diabetes Mellitus and Periodontal Disease

**DOI:** 10.1155/2014/917631

**Published:** 2014-11-30

**Authors:** Adriana Monea, Tibor Mezei, Sorin Popsor, Monica Monea

**Affiliations:** ^1^Department of Odontology and Periodontology, Faculty of Dental Medicine, University of Medicine and Pharmacy Târgu Mureș, 38 Gheorghe Marinescu Street, 540114 Târgu Mureș, Romania; ^2^Department of Morphopathology, Faculty of Medicine, University of Medicine and Pharmacy Târgu Mureș, 38 Gheorghe Marinescu Street, 540114 Târgu Mureș, Romania; ^3^Department of Prosthetics and Oral Rehabilitation, Faculty of Dental Medicine, University of Medicine and Pharmacy Târgu Mureș, 38 Gheorghe Marinescu Street, 540114 Târgu Mureș, Romania

## Abstract

*Objective*. To investigate oxidative stress (OS) and histological changes that occur in the periodontium of subjects with type 2 diabetes mellitus without signs of periodontal disease and to establish if oxidative stress is a possible link between diabetes mellitus and periodontal changes. *Materials and Methods*. Tissue samples from ten adult patients with type 2 diabetes mellitus (T2D) and eight healthy adults were harvested. The specimens were examined by microscope using standard hematoxylin-eosin stain, at various magnifications, and investigated for tissue levels of malondialdehyde (MDA) and glutathione (GSH). *Results*. Our results showed that periodontal tissues in patients with T2D present significant inflammation, affecting both epithelial and connective tissues. Mean MDA tissue levels were 3.578 ± 0.60 SD in diabetics versus 0.406 ± 0.27 SD in controls (*P* < 0.0001), while mean GSH tissue levels were 2.48 ± 1.02 SD in diabetics versus 9.7875 ± 2.42 SD in controls (*P* < 0.0001). *Conclusion*. Diabetic subjects had higher MDA levels in their periodontal tissues, suggesting an increased lipid peroxidation in T2D, and decreased GSH tissue levels, suggesting an alteration of the local antioxidant defense mechanism. These results are in concordance with the histological changes that we found in periodontal tissues of diabetic subjects, confirming the hypothesis of OS implication, as a correlation between periodontal disease incidence and T2D.

## 1. Introduction

In the recent years, an increasing number of scientists studied oxidative stress (OS) as a possible link between oral and systemic diseases. Many of these studies are related to periodontal disease, by far the most common link being oral infection [1].

Oxidative stress plays a major role in the pathogenesis of many systemic and oral diseases [2]. There is substantial evidence that links some oral diseases, such as periodontal disease, to systemic conditions such as cardiovascular diseases, metabolic syndrome, or diabetes mellitus [1]. Diabetes mellitus, a chronic disease, considered epidemical by World Health Organization (WHO), is by now recognized as a risk factor for periodontal disease. Also, periodontal disease was stated the 6th complication of diabetes mellitus. Reactive oxygen species (ROS) or free oxygen radicals are products of normal cellular metabolism and are produced in case of oxidative processes. Many biochemical pathways strictly associated with hyperglycemia, such as glucose autooxidation, polyol pathway, prostanoid synthesis, and protein glycation, can increase ROS production. Furthermore, endothelial cell exposure to elevated levels of glucose can lead to ROS production [3]. This might be one of the reasons why alterations of periodontal tissues occur in type 2 diabetes subjects (T2D), even in the absence of dental plaque and calculus which are the main etiologic factors of periodontal disease.

## 2. Objective

The aim of this study was to investigate oxidative stress that occurs in the periodontium of subjects with type 2 diabetes mellitus without signs of periodontal disease and to establish a possible link between this systemic condition and the morphologic changes in periodontal structures.

## 3. Material and Methods

The present study was conducted on two groups of patients; one consisted of ten diabetic patients without signs of periodontal disease and the other one consisted of eight systemically and periodontally healthy subjects as controls. Diabetic subjects were recruited from the patients that addressed to the Department of Odontology and Periodontology, Faculty of Dental Medicine, UMF Tg-Mures, for different dental problems, and controls were selected from the patients that addressed to the Emergency Department, Faculty of Dental Medicine, UMF Tg-Mures, for the same reasons. All diabetic subjects included in the present study had records of at least 4 to 5 years of diagnosed T2D, were on medication with oral antidiabetic drugs, and all had well-controlled T2D; none was insulin treated and none used any antioxidant agent. Subjects aged 30 to 58, nonsmokers, without any inflammatory disease or use of anti-inflammatory drugs in the last three months prior to the study.

This study was approved by the Ethical Committee of UMF Tg-Mures, and all the patients included in the study signed for informed consent.

From each subject biopsy specimens were obtained during the extraction of irrecoverable teeth. Biopsy specimens were harvested from a dental-periodontal unit in the posterior region of dental arches. From the biopsy specimens harvested from all subjects, one fragment was sent for histopathology study, while the other was stored at −80°C until OS evaluation.

Histopathological examination was performed using formalin-fixed, paraffin-embedded tissue fragments following standard protocols. The 4-5 micron thick tissue sections were stained with hematoxylin-eosin stain and also digitally archived using Zeiss MiraxScan system.

From conserved tissue biopsies we determined the levels of malondialdehyde (MDA) as a marker for OS and glutathione (GSH) as a marker of defense antioxidant mechanism, using the fluorometric methods according to Conti et al. [4] and Ellman [5]. The results were expressed in nmol/mg protein.

Statistical analysis was performed using Microsoft Excel, Graph-Pad InStat, and NCSS 2007 software, and values of *P* < 0.05 were considered statistically significant.

## 4. Results

Mean age of patients in study group was 44.7 ± 7.66 and 44.125 ± 6.728 in controls, with a nonsignificant difference between groups (*P* = 0.8696).

**Figure 1:**
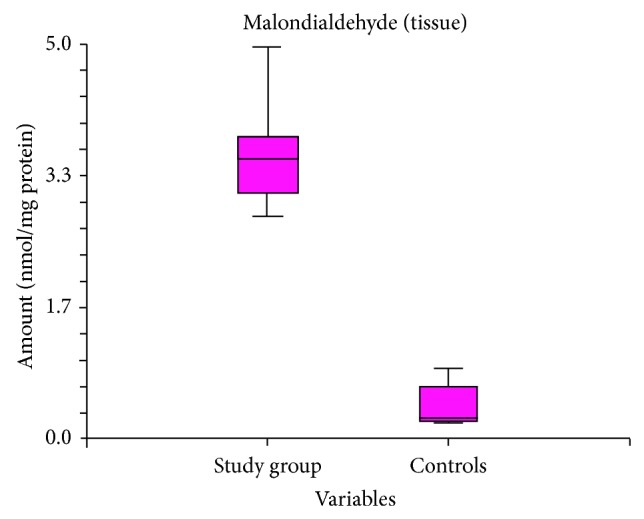
Comparison between MDA tissue levels in diabetics versus controls.

The mean MDA value in diabetic tissues was 3.578 (2.83–4.960), significantly higher than in controls, where the mean MDA value was 0.406 (0.21–0.9). Statistical comparison between the two groups yielded a *P* < 0.0001 (Figure 1).

**Figure 2:**
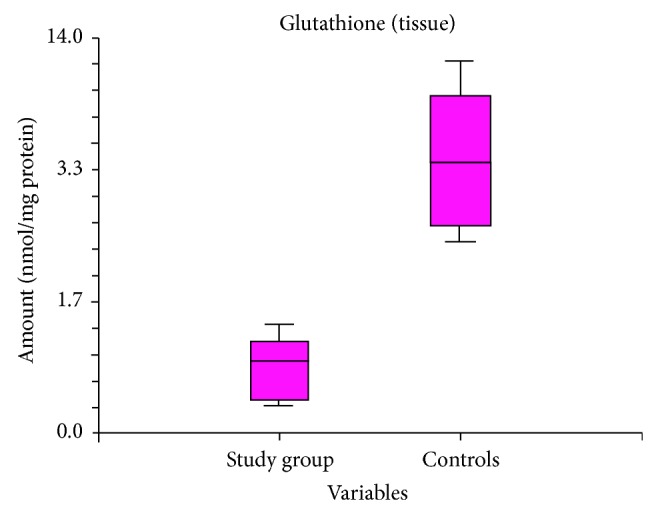
Comparison between GSH tissue levels in diabetics versus controls.

The mean GSH value in diabetic subjects was 2.48 (0.98–3.9), significantly lower than 9.788 (6.8–13.2) in controls, with *P* < 0.0001 (Figure 2).

**Figure 3:**
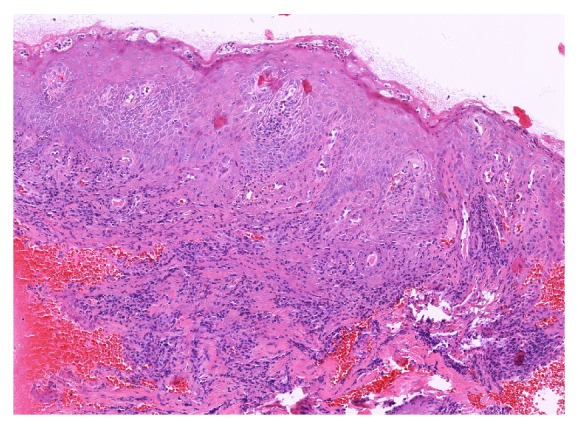
Inflammation within the epithelium and the lamina propria (H&E stain, 10x).

**Figure 4:**
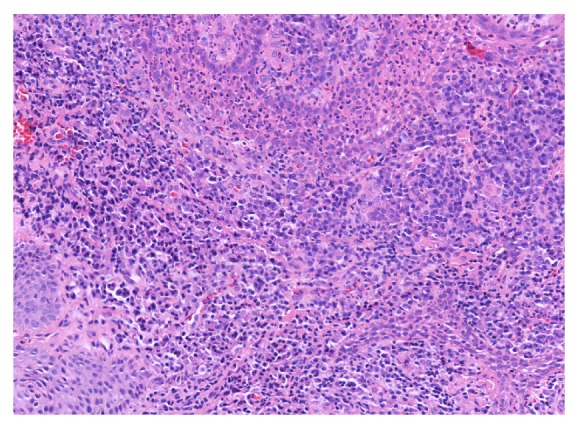
Polymorphic inflammatory infiltrate, predominantly chronic in the lamina propria and with granulocytes within the epithelium (H&E stain, 20x).

Histological alterations in tissue sections obtained from diabetic patients were present in both the epithelium and the lamina propria of the gingival mucosa. The epithelium displayed variable amounts of acanthosis and parakeratosis, with reduced quantities of acute inflammatory infiltrate composed mostly of polymorphonuclear leucocytes (segmented granulocytes) throughout its thickness and in superficially located microabscesses. A diffuse polymorphous inflammatory infiltrate consisting of lymphocytes, plasma cells, and, to a lesser extent, granulocytes was present in the mildly fibrotic lamina propria, displacing collagen fibers and surrounding ectatic blood vessels and exteriorized erythrocytes (Figures 3 and 4).

## 5. Discussions

The hypothesis of the study was that diabetes mellitus can increase OS at periodontal tissues level, contributing to periodontal disease development. Our results showed that tissue MDA and GSH levels differ significantly in diabetics without signs of periodontal disease compared to controls. In our study, tissue levels of MDA were higher in diabetics than in controls. As MDA (the final result of lipid peroxidation) is considered a biomarker of OS [6], elevated tissue MDA levels can support the hypothesis of OS implication in pathogenesis of periodontal disease in diabetic patients. Glutathione is a tripeptide present in almost all human cells. It is considered the most important nonenzymatic antioxidant agent, having an important role in antioxidant defense mechanism and in regulation pathways that insure whole body homeostasis [7–9]. In our study we identified lower glutathione levels in tissues harvested from diabetics compared to controls. This can stem from an adaptation of glutathione turnover as a response to OS caused by systemic condition, namely, diabetes mellitus.

The microscopic changes are in concordance with previously published data [10] and with those of other authors, confirming the fact that the presence of a metabolic condition such as diabetes mellitus and associated hyperglycemia can induce alterations of marginal periodontal structures, increasing the chance of periodontal disease occurrence [11–15].

Oxidative stress (OS) can be defined as an imbalance between the production of some highly reactive molecule species and the antioxidant defense mechanism [2]. Reactive oxygen species (ROS) or free oxygen radicals are products of normal cellular metabolism; however, their unbalanced increased levels disrupt normal cellular function. The most common free oxygen radicals are hydroxyl (HO^−^), nitric oxide (NO), superoxide (O_2_^−^), hydrogen peroxide (H_2_O_2_), and peroxynitrite (ONOO^−^). ROS can react with different amino acids, generating a large range of products, from modified and less reactive enzymes to denatured, nonfunctional proteins. An important structural modification of protein molecules is nitrosylation, with peroxynitrite being responsible for this change. Tyrosine is an important amino acid involved in phosphorylation reactions and signal transduction pathways. Tyrosine nitration not only compromises protein function but may also have serious consequences in cell regulation. Enzymes such as superoxide dismutase (SOD) were identified as specific targets for nitrosylation.

Several studies have shown a decreased superoxide dismutase activity in the gingival tissue of parodontopathic patients compared to parodontopathic diabetic patients. This can be a compensatory mechanism derived from hyperglycemia. The diabetic with periodontal disease has a lower activity of the prooxidative enzyme myeloperoxidase in the gingival crevicular fluid, compared to nondiabetics [16].

Reaction between mononuclear phagocytes and AGE (advanced glycation end products) induce the regulation of cytokine expression and OS [15]. These periodontal infections amplify the magnitude of macrophage response to AGE, leading to cytokine and OS production. This may explain the prevalence and severity of periodontitis in diabetic patients. Many studies have shown a decreased SOD activity in the gingival tissue of periodontotic patients compared to parodontotic diabetic patients. This can be a compensatory mechanism derived from hyperglycemia. Patients with diabetes and periodontal disease have a lower activity of the prooxidative enzyme myeloperoxidase in the gingival crevicular fluid, compared to nondiabetics [17]. Also, it was proven that there is a relationship between proinflammatory biomarkers and OS in case of periodontal disease [18].

The clinical relevance of our study is that MDA and GSH levels can predict the evolution of periodontal disease in subjects with type 2 diabetes mellitus.

Limitations of the study are that we included only nonsmokers to minimize confounding variables. Further research should include smokers with T2D and periodontal disease, as smoking is a common habit in Romanian population and its effect on oral mucosa needs to be taken into consideration.

One topic that remains to be explored is how antioxidant periodontal therapy can influence periodontal alterations in diabetic patients.

## 6. Conclusions

Diabetic subjects have higher MDA levels in their periodontal tissues, suggesting an increased lipid peroxidation in case of diabetes mellitus. In periodontal tissues from diabetics, decreased levels of glutathione suggest an alteration of local antioxidant defense mechanism. These results are in concordance with the histological changes that we found in periodontal tissues of diabetic subjects. The results of our study confirm the hypothesis of OS implication, as a correlation between periodontal disease incidence and diabetes mellitus. These results can be a starting point for further research on the efficiency of different antioxidant agents for prevention and treatment of periodontal disease in diabetic patient.
